# Exacerbation of symptomatic arthritis by cigarette smoke in experimental arthritis

**DOI:** 10.1371/journal.pone.0230719

**Published:** 2020-03-27

**Authors:** Jaewoo Kang, Sang Hoon Jeong, Kijun Lee, Narae Park, Hyerin Jung, Kyuhong Lee, Ji Hyeon Ju

**Affiliations:** 1 CiSTEM laboratory, Catholic Induced Pluripotent Stem Cell (iPSC) Research Center, College of Medicine, The Catholic University of Korea, Seoul, Republic of Korea; 2 Department of Biomedicine & Health Science, Seoul St. Mary’s Hospital, College of Medicine, The Catholic University of Korea, Seoul, Republic of Korea; 3 Jeonbuk Department of Inhalation Research, Korea Institute of Toxicology, Jeongeup, Jeollabuk-do, Republic of Korea; 4 Division of Rheumatology, Department of Internal Medicine, College of Medicine, Seoul St. Mary’s Hospital, The Catholic University of Korea, Seoul, Republic of Korea; US Department of Veterans Affairs, UNITED STATES

## Abstract

**Introduction:**

Epidemiologically, cigarette smoking is a well-known risk factor for the pathogenesis of rheumatoid arthritis (RA). However, there has been few plausible explanations why cigarette smoking aggravated RA. We investigated the causal effect of smoking in experimental model of arthritis development.

**Methods:**

During induction of experimental arthritis with collagen challenge, mice were exposed to a smoking environment with 3R4F cigarettes. Generated smoke was delivered to mice through a nose-only exposure chamber (ISO standard 3308). Human cartilage pellet was challenged by cigarette smoke extract to identify citrullinating potential *in vitro*.

**Results:**

Cigarette smoke exacerbated arthritis in a collagen-induced arthritis (CIA) model. Exposure to smoke accelerated the onset of arthritis by 2 weeks compared to the conventional model without smoke. Citrullination of lung tissue as well as tarsal joints were revealed in smoke-aggravated CIA mice. Interestingly, tracheal cartilage was a core organ regarding intensity and area size of citrullination. The trachea might be an interesting organ in viewpoint of sharing cartilage with joint and direct smoke exposure. Anti-CCP antibodies were barely detected in the serum of CIA mice, they were significantly elevated in cigarette smoke group. Citrullinated antigens were increased in the serum of smoke-exposed mice. Lastly, a cigarette smoke extract enhanced human cartilage citrullination *in vitro*.

**Conclusions:**

Missing link of arthritic mechanism between smoke and RA could be partially explained by tracheal citrullination. To control tracheal cartilage citrullination may be beneficial for preventing arthritis development or aggravation if cigarette smoke is becoming a risk factor to pre-arthritic individual.

## Introduction

Rheumatoid arthritis (RA) is an autoimmune disease characterized by synovial inflammation, autoantibody production, and joint destruction [1]. The exact mechanism of the pathogenesis of RA is unclear; however, several factors including genetics and environmental factors such as viral infection and cigarette smoking are considered to contribute to the development of RA [2–4].

Citrullination is a major type of post-translational protein modification that is frequently observed in RA. Arginine is converted to citrulline through the enzymatic activity of peptidylarginine deiminases (PADs) [5]. Several genetic and environmental factors contribute to the overexpression of PAD in RA. PAD4 is a calcium dependent enzyme and displays positive cooperativity. High concentrations of calcium ions lead to cellular PAD activation under inflammatory conditions, which in turn leads to abnormal production of citrullinated proteins [6–8]. Especially, PAD2 and PAD4 are linked to citrullination of synovial tissues. The constituent proteins of synovial membrane (i.e. vimentin, collagen, and fibronectin) are easily citrullinated, leading to increased levels of anti-CCP antibodies [9–11].

Cigarette smoking is known as an important risk factor for the pathogenesis of RA [12]. Cigarette smoking increases levels of inflammation, oxidative stress, and hypoxic conditions [2], and heavy smokers have a higher risk of developing RA compared to non-smokers [13]. Cigarette smoking has also been implicated as a cause of RA in many case-control studies over the years [14, 15]. Especially, the respiratory tract can be directly damaged by cigarette smoke which leads to pulmonary complications in RA patients. RA patients in turn can develop RA associated-interstitial lung disease (RA-ILD) as well as several other pulmonary disorders; usual interstitial pneumonia (UIP), bronchiolitis, and rheumatoid nodules [16–18].

12G1, the novel anti-citrullinated protein antibody (ACPA) that was developed by injecting cyclic citrullinated antigen in mice and hybridized with B cells producing citrullinated peptide antibodies with a myeloma cell line enables detecting citrullinated proteins in CIA mice. The novel ACPA is citrulline-specific monoclonal antibody that utilizes various methods. 12G1 shows high specificity to target proteins that are prone to being citrullinated [19]. Citrullinated proteins are known for their involvement in the pathogenesis of RA [20]. 12G1 is useful tool that identifies citrullinated proteins of target proteins, including vimentin [21], enolase [22], fibronectin [23] and collagen II [24].

In this study, we investigated the role of cigarette smoke in a mouse model of collagen-induced arthritis (CIA). We recognized that standardizing smoke exposure was essential for accurate analysis of the direct arthritic effect of smoking. Thus, CIA mice were exposed to inhaled cigarette smoke in a GLP facility specializing in toxicology.

## Methods

### Ethical approval

All procedures on experimental animals were performed in accordance with the Laboratory Animals Welfare Act, the Guide for the Care and Use of Laboratory Animals, and the Guidelines and Policies for Rodent Experiments provided by the Institutional Animal Care and Use Committee in the School of Medicine, The Catholic University of Korea. This study protocol was approved by the Institutional Review Board of The Catholic University of Korea (CUMC-2018-0055-11).

### Induction and evaluation of collagen-induced arthritis

Six-week-old female DBA/1J mice (OrientBio, Seongnam, Korea) maintained under pathogen-free conditions. Mice were subdivided into 4 groups and each group was composed of 5 mice. Mice were immunized by intradermal injection at the base of the tail at day 0. Each mouse was injected intradermally with using 2mg/ml Bovine type II collagen (CII; Chondrex, Redmond, WA, USA) and 2mg/ml Complete Freund’s adjuvant (CFA; Chondrex, Redmond, WA, USA) emulsion. The emulsion was made before immunization of equal volume of CII/CFA and injected with 100μl to each mouse. After immunization, CIA symptoms were scored as an arthritic score based on the severity of swollen paws in a blinded manner. Development of arthritis was measured two times per week and scored from 0 to 4(0, normal; 1, swelling of a toe and mild swelling to the tarsals; 2, swelling of two or more toes or joints and increased swelling; 3, erythema and moderate swelling in the tarsal joints; 4, definite erythema with severe swelling encompassing the ankle, foot, and digits) [25]. The arthritic score was combined score of all four paws. The arthritis incidence was calculated as the percentage of swollen paws in each mouse.

### Cigarette smoke exposure

Normal control mice group was exposed to filtered clean air while experimental mice groups were exposed to smoke from 3R4F cigarettes (Tar 9.4mg/cigarette, nicotine 0.73mg/cigarette). Exposures were for one hour per day 5 days per week for 4 weeks after CII/CFA immunization. Cigarette smoke was generated with a cigarette smoke generator with a puff volume of 35ml per puff, duration of 2 seconds per puff, and interval of 60 seconds [ISO standard 3308: Routine analytical cigarette-smoking machine—Definitions and standard conditions (2000)]. Experimental mice groups were divided into low cigarette smoke concentration (150μg/L) and high cigarette smoke concentration (600μg/L) and administered in a nose-only exposure chamber.

### Preparation of cigarette smoke extract

Cigarette smoke extract (CSE) was prepared as described previously [26]. Briefly, one cigarette (Marlboro red, Phillp Morris, 0.7mg nicotine and 8mg tar) was bubbled through 10ml PBS with a vacuum pump. The cigarette smoke extract was then filtered through a 0.22μm. Culture medium was prepared to dilute the CSE to the desired concentration.

### Histological analysis

All mice were anesthetized by isoflurane to minimize animal suffering and sacrificed by CO_2_ inhalation. After sacrifice, the hind limb was fixed and decalcified in 10% EDTA. The tissues were embedded in paraffin blocks and sectioned. Prepared slides were stained with hematoxylin and eosin (H&E), safranin O, and toluidine blue. The inflammation score and joint destruction score were measured by three researchers in a blinded manner as described by Huckel *et al* [27]. Briefly, the inflammation score was calculated as the severity of infiltration and pannus formation and the destruction score was calculated as the extent of cartilage and bone destruction. For alcian blue staining, sections were incubated in a 1% alcian blue solution and counterstained with nuclear fast red.

### Immunohistological staining and immunofluorescence

To confirm the presence and extent of citrullinated proteins, anti-CCP antibody (12G1, AR13-MA0001, Abfrontier, Seoul, Korea) was used. All slides were blocked endogenous peroxidase activity by 3% H_2_O_2_. After washing, non-specific antibody binding was blocked with the mouse IgG blocking agent in Mouse on Mouse (M.O.M) Basic Kit. A 12G1 antibody was diluted in M.O.M diluent solution and incubated on slides overnight at 4°C. The next day, the slides were incubated with Biotinylated Anti-Mouse Ig Reagent and an avidin-biotin complex using VECTASTAIN Elite ABC HRP Reagent R.T.U. The DAB Peroxidase (HRP) Substrate Kit was used for chromogenic reactions, followed by counterstaining with mayer’s hematoxylin.

To confirm co-localization with synovial proteins in tissues, anti-vimentin antibody (sc7558, Santa Cruz, CA, United States) and 12G1 antibody were used for immunofluorescence staining. After blocking, slides were incubated by M.O.M blocking Reagent. 12G1 antibody was diluted in M.O.M diluent solution and incubated overnight at 4°C. Next, the slides were incubated with Biotinylated Anti-Mouse Ig Reagent and incubated with Fluorescein Avidin DCS. For blocking, 10% normal goat serum containing 1% PBA (PBS+1% bovine serum albumin) at RT. Anti-vimentin antibody was diluted in 5% normal goat serum containing 1% PBA and incubated overnight at 4°C. Alexa Fluor 594 goat anti-rabbit IgG (H+L) antibody was diluted in PBS and incubated at RT. Nuclear staining was performed using 4', 6-diamidino-2-phenylindole (DAPI).

### Enzyme linked immunosorbent assay (ELISA)

To analyze the amount of cyclic citrullinated proteins, serum was isolated from mice blood. Prior to starting the assay, each antibody (collagen type Ⅱ, vimentin, enolase, filaggrin, fibronectin) was coated on plates at 4°C overnight. The next day the plates were washed in wash buffer containing 0.05% tween 20 in PBS and blocked with 5% skim milk dissolved in ELISA assay buffer for 1 hour at RT. After blocking and washing, each group of serum samples added and incubated for 2 hours at RT. The plates were then washed and re-blocked as above. After washing again, 12G1, an anti-CCP antibody was used as the capture antibody and incubated for 2 hours at RT. After washing, a goat anti-mouse IgG antibody was incubated for 1 hour at RT. The plates were then washed, and TMB solution was added for 15 minutes under blocking light. A stop solution (0.16M sulfuric acid) was added and the absorbance was measured at 450nm. In addition, the amount of anti-CCP antibody in serum was analyzed using a mouse anti-cyclical citrullinated peptide antibody (ACPA) ELISA kit (Mybiosource, San Diego, CA, USA) according to the manufacturer’s recommended protocol.

### Cell culture

Cord blood mononuclear cell (CBMC)-derived induced pluripotent stem cells (iPSCs) were established and characterized as described previously [28]. In our previous study, we established 13 CBMC-derived iPSC cell lines, characterized by high pluripotency and normal karyotypes. Briefly, for chondrogenic differentiation, iPSCs were resuspended in Aggrewell medium (Stemcell) to induce embryonic bodies (EBs) and the media was changed to Essential 8 medium. EBs were resuspended in DMEM supplemented with 20% fetal bovine albumin (FBS) to induce outgrowth cells. After induction, 3 x 10^5^ cells per pellet were resuspended in chondrogenic differentiation medium and transferred to 15ml conical tubes. The resulting pellets were maintained for 30 days for maturation [29]. Peripheral blood mononuclear cells (PBMCs) were incubated in RPMI-1640 medium supplemented with 10% FBS, and chondrogenic pellets were co-cultured with PBMCs exposed to normal media or media containing cigarette smoke extract.

### Statistical analysis

Statistical analysis was performed using GraphPad Prism 5 (GraphPad). Results are shown as the mean and standard error of the mean. Error bars represent the standard error of the mean. Comparison of multiple groups were analyzed by one-way ANOVA followed by Tukey’s *ad hoc* test to determine significant differences. Arthritis scores were compared using a repeated statistical test. *p*-values *<*0.05 were considered to indicate statistical significance.

## Results

### Cigarette smoke exacerbates symptomatic arthritis in a collagen-induced arthritis (CIA) mouse model

**Fig 1:**
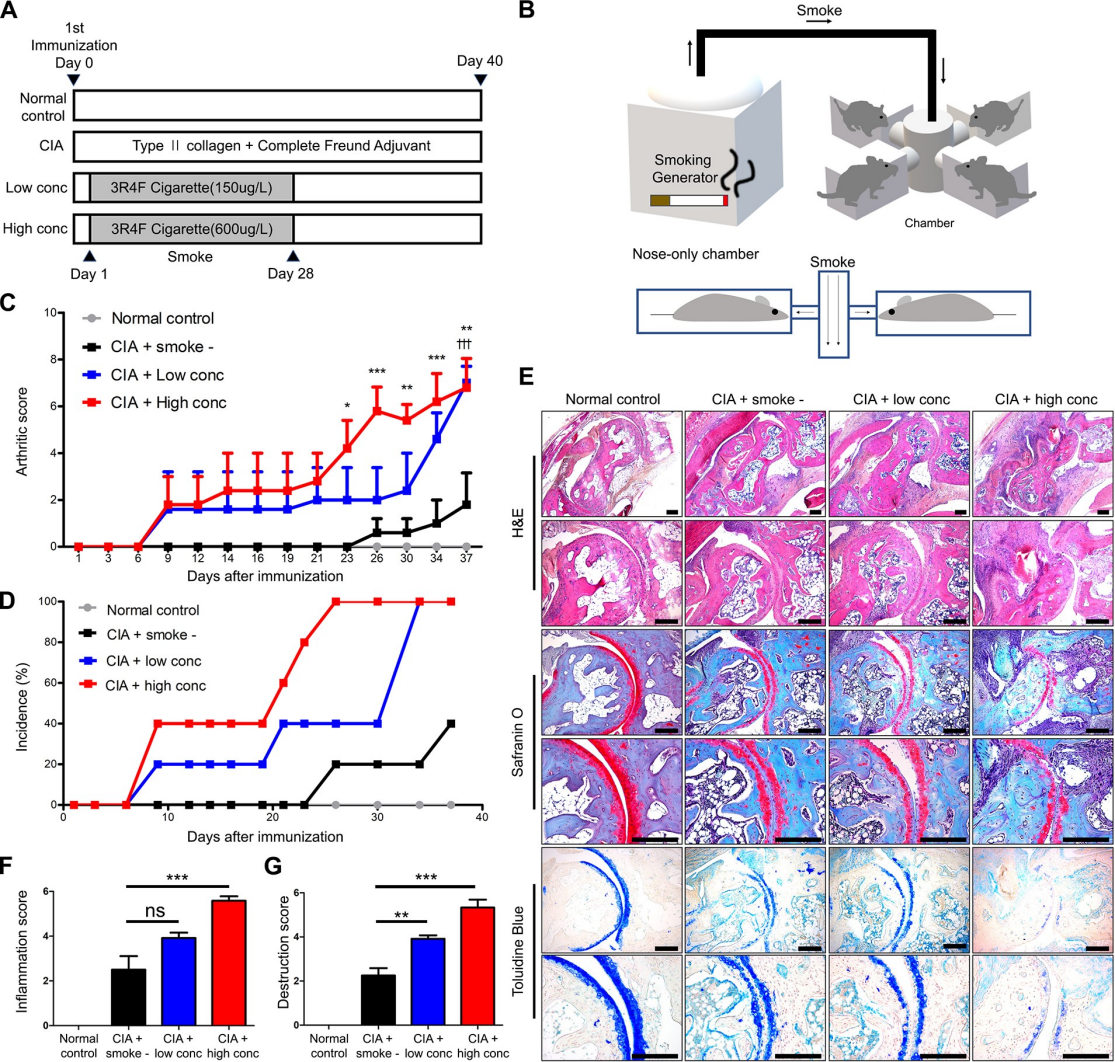
Cigarette smoke exacerbates rheumatoid arthritis in a collagen-induced arthritis (CIA) mouse model. (A) Scheme of the experimental concept of CIA induction and cigarette smoke inhalation. (B) Schematic diagram of cigarette smoke inhalation chamber. CIA mice were placed in a nose-only exposure smoke chamber. All mice were made to inhale cigarette smoke for one hour per day for 4 weeks. (C) The arthritic score was calculated 2 times per week in the experimental period. Using established criteria, each paw was given an arthritic score that ranged from 0 to 4, and the scores from all 4 paws were summed. (D) The incidence of arthritis was calculated according to the percentage of swollen and inflammatory paws of each mouse. (E) Histological analysis was conducted by H&E, safranin O, and toluidine blue staining of the tarsal joint in the hind paws. (F) Inflammation score showed the extent of synovial hyperplasia and infiltration of immune cells such as leukocytes. (G) Destruction score showing loss of cartilage according to safranin O and toluidine blue staining. Histological scores were calculated by three researchers in a blinded manner. Data are the mean±SEM, †††*p*<0.001, CIA versus CIA with low concentration of smoke, *p<0.05; **p<0.01; ***p<0.001, CIA versus CIA with high concentration of smoke by repeated statistical analysis and multiple groups by one-way ANOVA. CIA, collagen-induced arthritis. Scale bars, 200μm.

The experimental mice were divided into CIA mice with low cigarette smoke concentration (150μg/L) and high cigarette smoke concentration (600μg/L) groups (Fig 1A). A schematic diagram in Fig 1B showed the process for generating cigarette smoke and placement of mice in the nose-only exposure smoke chamber. The arthritic score and arthritis incidence were higher in both cigarette smoke groups compared to the CIA group without smoke exposure (Fig 1C and 1D). Higher smoke concentration increased arthritic severity by five arthritis points than low smoke concentration. For histological analysis, tarsal joints of mice were stained with hematoxylin-eosin (H&E), safranin O and toluidine blue (Fig 1E). The CIA with cigarette smoke groups showed greater cellular infiltration and pannus formation, as well as higher degree of cartilage damage compared to other groups. Based on these histological analysis, inflammation and destruction score were calculated (Fig 1F and 1G). Both scores were significantly higher in the CIA mice exposed to a high concentration of cigarette smoke compared to both the low concentration and CIA groups.

### Citrullinated proteins are more prominent in CIA mice exposed to cigarette smoke compared to CIA mice

**Fig 2:**
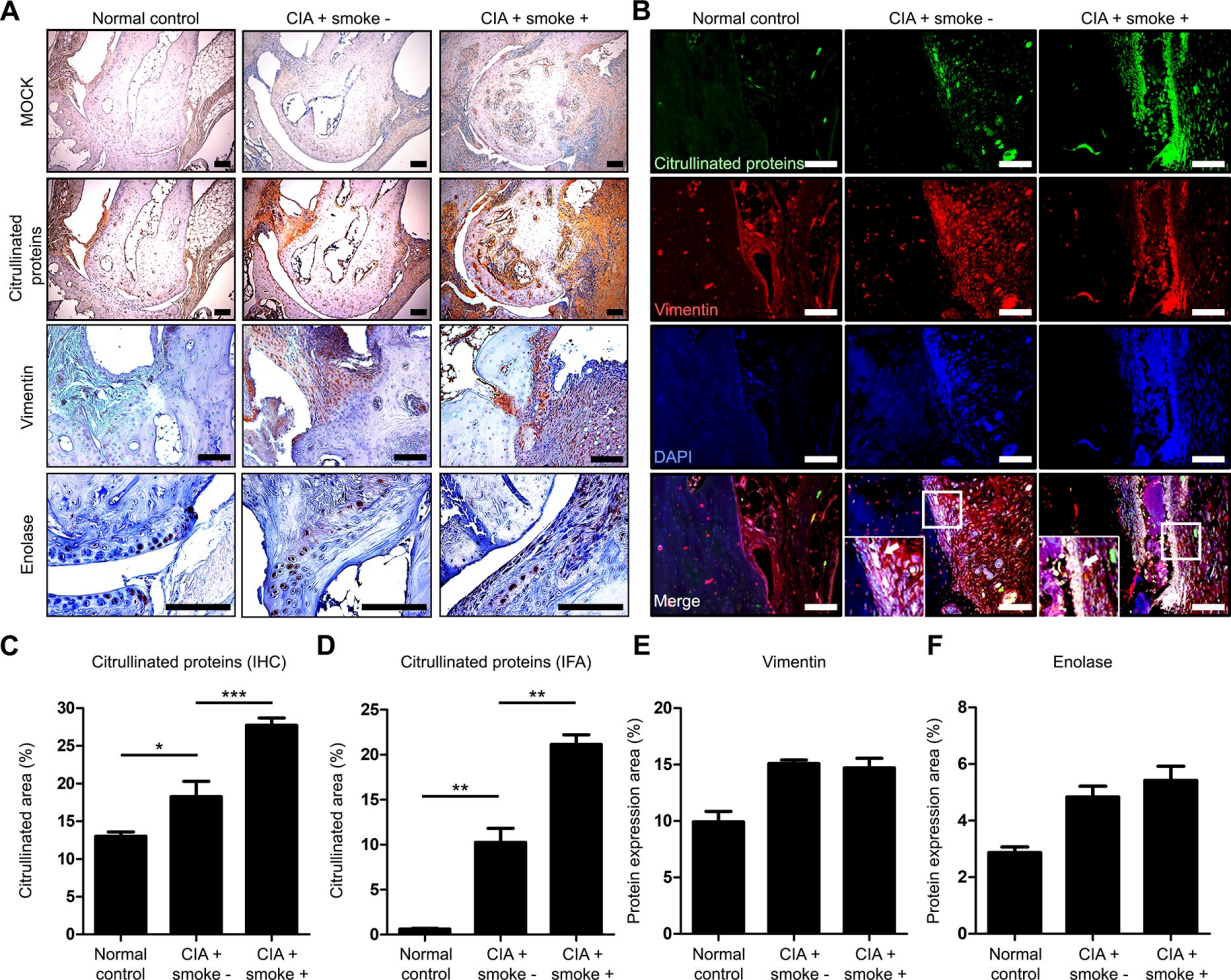
Citrullinated proteins are higher in the tarsal joints of CIA mice exposed to cigarette smoke compared to CIA mice. An anti-CCP antibody (12G1) was used to detect citrullinated proteins in the tarsal joints. (A) Immunohistochemical staining, we detected vimentin and enolase, which are prone to citrullination, and total citrullinated proteins by 12G1 staining in the tarsal joints of each group. (B) Immunofluorescence assay showed that vimentin and citrullinated proteins were co-localized in the tarsal joints of each group. (C, D) The citrullinated area was calculated by quantitative analysis. The positive area of (E) vimentin and (F) enolase was calculated. Data are the mean±SEM, *p<0.05; **p<0.01 by one-way ANOVA. CIA, collagen-induced arthritis; IHC, immunohistochemical staining; IFA, immunofluorescence assay; DAPI, 4', 6-diamidino-2-phenylindole. Scale bars, 100μm.

Citrullination is a key process in arthritis development [30]. Using immunohistochemical staining (IHC), we confirmed that citrullinated proteins were detected at a higher level in CIA mice exposed to cigarette smoke compared to the CIA group. Vimentin and enolase were intensely citrullinated (Fig 2A). To confirm that vimentin and enolase were target proteins of citrullination, we performed an immunofluorescence assay (IFA) to analyze co-localization of citrullinated proteins and vimentin and enolase (Fig 2B, S1 Fig). The area of citrullination by 12G1 in IHC was significantly larger in CIA with cigarette smoke group than CIA group (Fig 2C). In addition, the area of citrullination in IFA was larger in the CIA group exposed to cigarette smoke compared to the CIA group (Fig 2D). These data showed that vimentin was the target protein of citrullination in the tarsal joint. In addition, there was no difference in the expression of uncitrullinated vimentin and enolase between groups (Fig 2E and 2F).

### Cigarette smoke affects the respiratory tract in CIA mice

**Fig 3:**
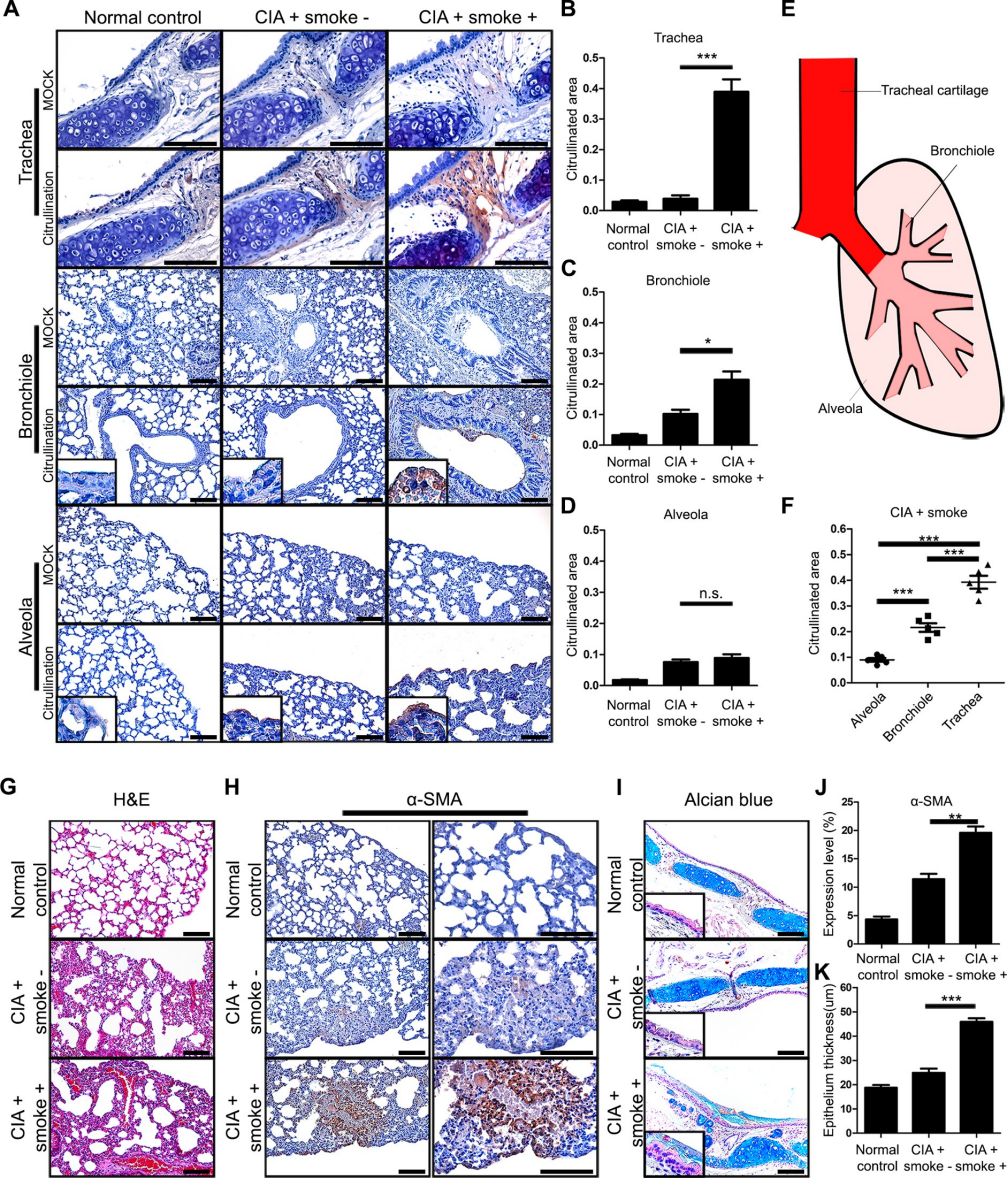
Cigarette smoke alters the respiratory tract in CIA mice. (A) Immunohistochemical staining was performed for tracheal, bronchiolar, and alveolar tissue. (B-D) The expression level of each tissue was quantified. (E) Schematic showing the hypothesized distribution of cigarette smoke reaching different parts of the lung. (F) Comparison of the expression level of each tissue. (G-I), Lung morphology shown by H&E staining and α-SMA protein expression by IHC. (J, K) Expression level of α-SMA and epithelium thickness was quantified. Data are the mean±SEM, *p<0.05; **p<0.01; ***p<0.001 by one-way ANOVA. CIA, collagen-induced arthritis; n.s., non-significant. Scale bars, 100μm.

We next prepared tissue of the trachea and lungs in longitudinal sections. Tracheal citrullination was most prominent in the CIA mice exposed to cigarette smoke group (Fig 3A). The citrullinated areas in the trachea, bronchioles, and alveola were calculated by quantitative analysis (Fig 3B–3D). Aerodynamic analysis showed that cigarette smoke particles reach more at respiratory tract as close to mouth (Fig 3E). It is assumed that more distant from mouth, less smoke reaches. In cigarette smoke group, the area of citrullination proteins in trachea was higher than that of bronchiole and alveola (Fig 3F). In addition, smoke exposure induced inflammatory cell infiltration and fibrosis (Fig 3G). Smoke also evoked more α-SMA fibrotic spots (Fig 3H). In the trachea, the thicknesses of the epithelium and mucus layers were increased in CIA mice exposed to smoke (Fig 3I). The expression level of α-SMA and thickness of epithelium were calculated by quantitative analysis (Fig 3J and 3K). Taken together, these results suggested that cigarette smoke damages the trachea and lungs by inducing fibrosis and citrullination, and that changes exacerbates arthritis.

### Cigarette smoke increases citrullinated proteins and anti-CCP antibodies in the serum of CIA mice

**Fig 4:**
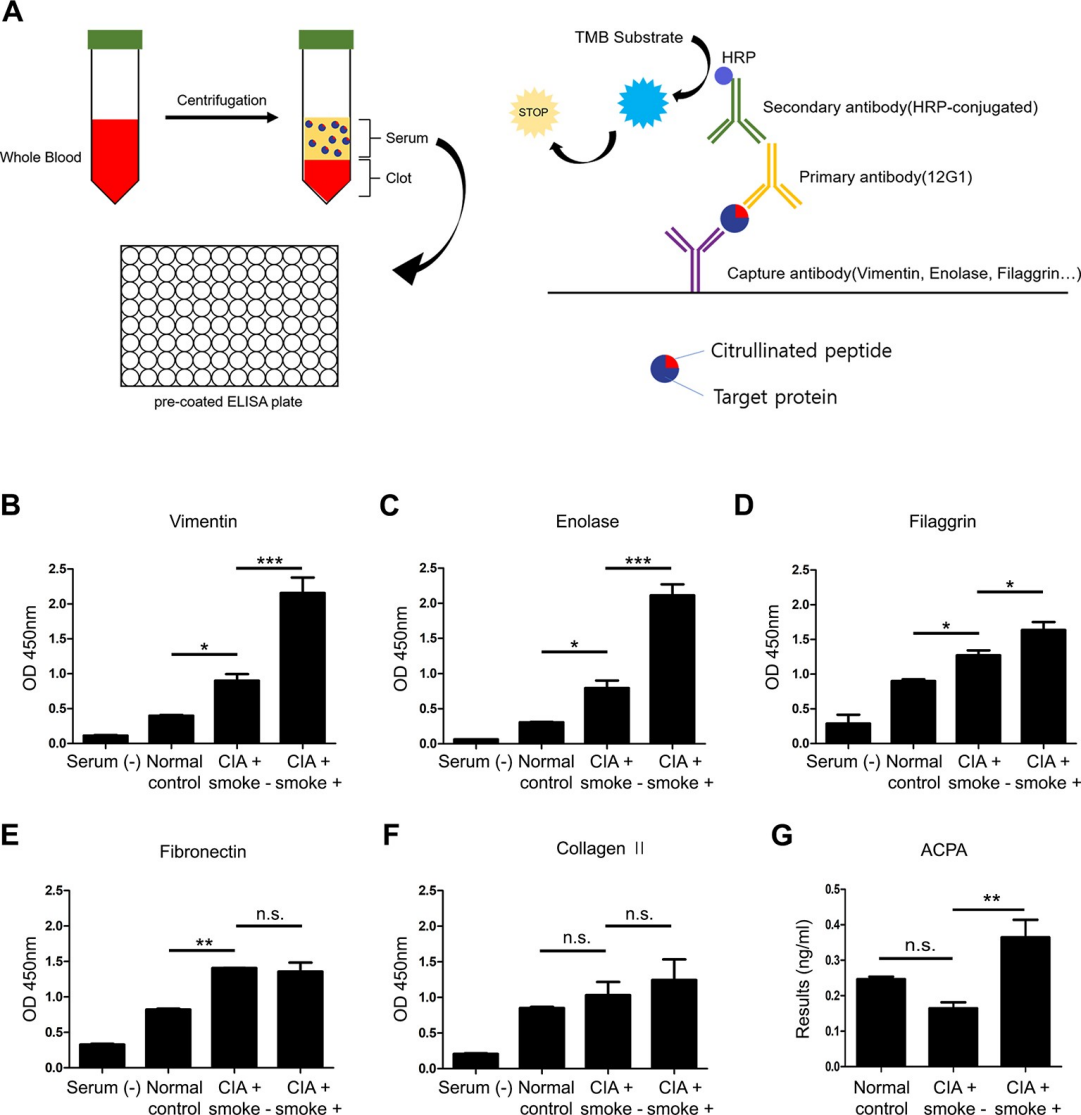
Serum levels of citrullinated proteins can be detected by a 12G1 antibody ELISA. (A) Diagram of the sandwich ELISA. The 12G1 antibody was used as the 1st antibody for detection of citrullinated proteins in ELISA. Levels of (B) vimentin, (C) enolase, (D) filaggrin, (E) fibronectin and (F) type II collagen detected by each antibody in mouse serum. Antibodies for each prospective protein were used as the capture antibody while the anti-CCP antibody was used as the detecting antibody. An HRP-conjugated detection antibody was used to evaluate anti-CCP antibody binding according to colorimetric change of the TMB substrate. After addition of the stop solution, absorbance was measured at 450nm. (G) Detection of anti-CCP antibody by ACPA ELISA kit. Data are the mean±SEM, *p<0.05; **p<0.01; ***p<0.001 by one-way ANOVA. OD, optical density; CIA, collagen-induced arthritis; ACPA, anti-citrullinated proteins antibody; n.s., non-significant.

After sacrifice, serum was isolated from mouse blood. We evaluated the amount of citrullinated proteins by ELISA with 12G1. To confirm the presence of citrullinated proteins in serum, we performed sandwich ELISA following the schematic diagram shown in Fig 4A. The target proteins, vimentin, enolase, filaggrin, Bip, type II collagen are prone to citrullination. The detection level of citrullinated vimentin, enolase, filaggrin was higher in the CIA mice exposed to cigarette smoke group than the CIA group (Fig 4B–4D). However, there was no significant difference in the level of citrullinated fibronectin and type II collagen between CIA and CIA mice exposed to smoke group (Fig 4E and 4F). In addition, the level of the anti-CCP antibody was higher in the CIA group exposed to smoke than the CIA group (Fig 4G). These results demonstrated that the citrullinated target proteins were more abundant in the CIA mice exposed to smoke.

### Cigarette smoke extract influences citrullination of cartilage pellets

**Fig 5:**
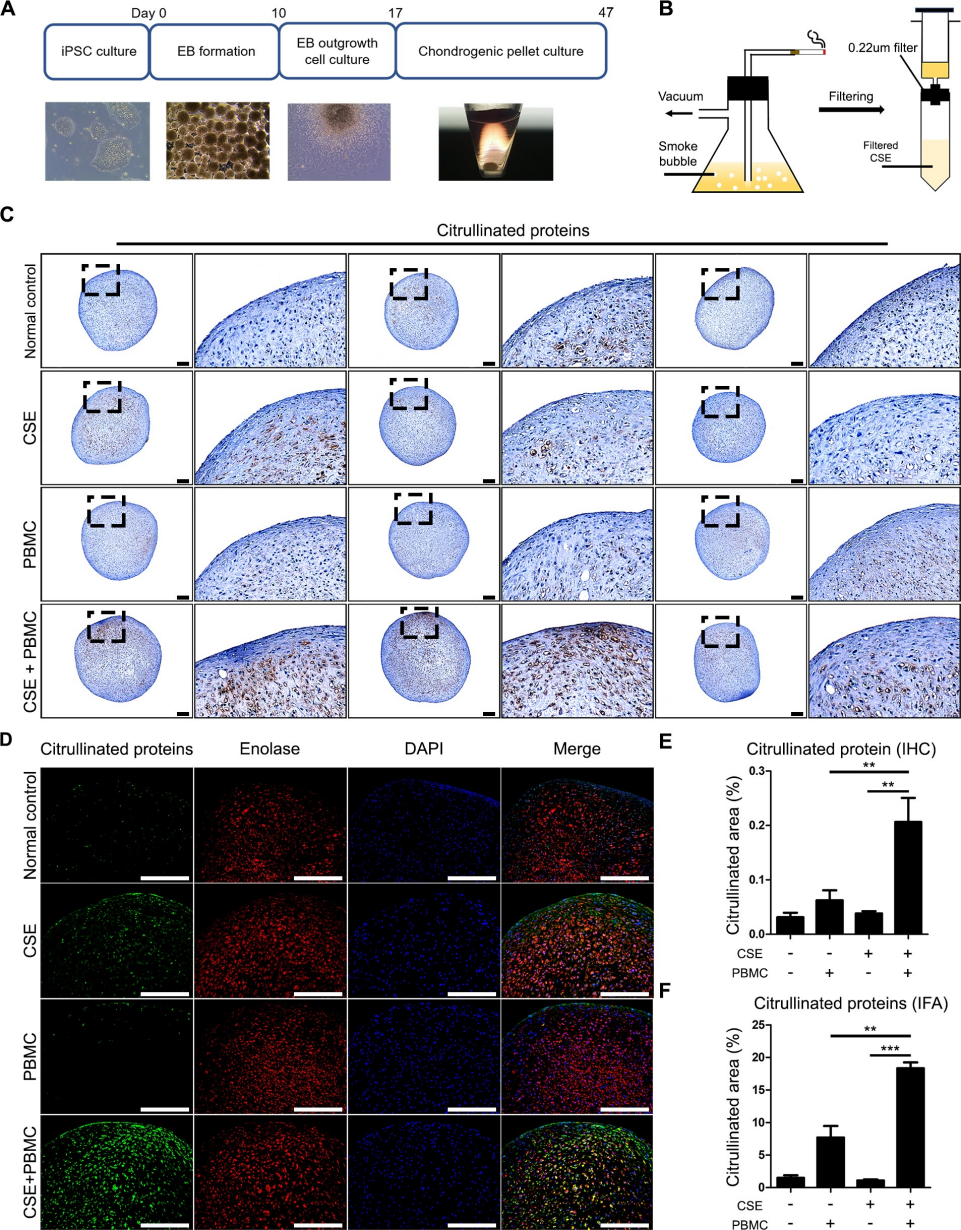
Cigarette smoke extract affects citrullination of iPSC-derived chondrogenic pellets. (A) Schematic diagram of chondrogenic pellets from iPSCs for each stage of chondrogenic differentiation. (B) Schematic diagram of cigarette smoke extract preparation. Cigarette smoke was dissolved in PBS and filtered sterilized with a 0.22μm filter before treatment. (C) Immunohistochemical staining was performed to confirm citrullinated proteins in chondrogenic pellet. (D) Immunofluorescence Assay showing co-localization of enolase and citrullinated proteins in chondrogenic pellet. (E, F) The citrullinated area was calculated. Data are the mean±SEM, **p<0.01; ***p<0.001 by one-way ANOVA. CSE, cigarette smoke extract; PBMCs, peripheral blood mononuclear cells. IHC, immunohistochemical staining; IFA, immunofluorescence assay; DAPI, 4', 6-diamidino-2-phenylindole. Scale bars, 200μm.

To confirm our observation that cigarette smoke increases citrullination, we prepared a cigarette smoke extract and generated cartilage pellets from human induced pluripotent stem cells. A scheme of the generation of cartilage pellets and morphology of each step of differentiation is shown in Fig 5A. A schematic diagram of making cigarette smoke extract was shown in Fig 5B. The cartilage pellets were co-cultured with cigarette smoke extract and peripheral mononuclear cells.

We conducted CCK-8 assays to identify the ideal concentration of cigarette smoke for the chondrogenic pellets (S2 Fig). Cultured cartilage pellets were stained by anti-CCP antibody. IHC revealed that co-culture with cigarette smoke extract resulted in a larger area of citrullination compared to control groups (Fig 5C). We also confirmed the presence of co-localization of citrullinated proteins and enolase (Fig 5D). The percent area of citrullination was also calculated (Fig 5E). Additionally, we found citrullination in vimentin peptide (LQEEMLQREEAENTLQSFR) by mass spectrometry (S3 Fig). Thus, we confirmed that cigarette smoke extract affected the extracellular matrix of chondrogenic pellets by increasing citrullination.

## Discussion

Rheumatoid arthritis is an autoimmune disease in which autoantibodies attack organs, especially joint cartilage as demonstrated by joint destruction and infiltration of immune cells [31]. Many factors influence the pathogenesis of RA. Similar to other autoimmune diseases, cigarette smoke is one of the most harmful factors in the pathogenesis of not only RA [32], but is also an important environmental factor in a variety of diseases [33]. The mechanism of diseases affected by cigarette smoke is not fully elucidated, but various studies showed bad effects of cigarette smoke.

Previous studies have shown that cigarette smoke is related to increased risk of RA development. Aryl hydrocarbon receptor (AHR), the transcription factor of the generation of Th17 cells is affected by cigarette smoke. AHR activation links the expansion of Th17 cells and the production of IL-17A, which is a pro-inflammatory cytokine that aggravates arthritis [34, 35]. Also, cigarette smoke is closely related to innate immunity. One of the defense mechanisms of the immune system is neutrophil extracellular traps (NET) formation that forms a net-like structure by the flow out the nuclear contents of neutrophils. The contents include citrullinated histone complexes, which is done by PAD4. Smoking induces NET formation that contributes to citrullination of the arginine residue in the extracellular histone complexes. Abnormally large amounts of citrullinated histone complexes contributes to the generation of ACPAs, which plays an important role in the pathogenesis of RA [36–40].

In the present study, we found that cigarette smoke exacerbates RA in a collagen-induced arthritis mouse model and increases the amount of citrullinated proteins and anti-CCP antibody levels in serum. We found evidence of excessive citrullinated proteins in numerous organs due to cigarette smoke.

In this study, we used a CIA mouse model [25] to evaluate the effects of cigarette smoke on arthritis using an arthritic score. The CIA experimental groups exposed to cigarette smoke showed higher arthritic scores and increased incidence of arthritis compared to the CIA group. Cigarette smoke promoted earlier onset of arthritis and increased severity of arthritis in a dose-dependent manner. CIA mice exposed to a low concentration of cigarette smoke showed significant differences of arthritic score compared to high concentration group only at intermediate timepoints, but reached similar final severity scores at the end of the experimental period (Fig 1C and 1D). The severity of arthritis was also evaluated by histological analysis using various stains. Consistent with the above results, high concentrations of cigarette smoke group damaged tarsal joints more than low concentration cigarette smoke and the CIA group (Fig 1E).

Vimentin is an intermediate filament protein that is involved in the organization of cytosolic organelles. Vimentin plays an important role in supporting cellular structure, regulating immune responses, and promoting wound healing [41, 42]. In addition, enolase is expressed in most tissues and plays a role as a glycolytic enzyme and cell surface receptor of plasminogen [43]. IHC showed that while expression of vimentin and enolase were not significantly altered by exposure to cigarette smoke, levels of the citrullination of both proteins were increased in CIA mice exposed to cigarette smoke compared with CIA group (Fig 2A). Citrullination was also analyzed by IFA that showed co-localization of vimentin and citrullinated proteins (Fig 2B). We used 12G1, a novel ACPA, for the detection of citrullinated proteins. ACPA is an important diagnostic factor for RA, in this regard, 12G1 can be a valuable agent to detect citrullinated proteins that produces ACPAs in human serum. The 12G1 is useful marker for RA diagnosis in ELISA method. Kim et al developed a novel ACPA, 12G1, which was reported as a useful agent for RA diagnosis through ELISA [19] and Ju and Kim developed an kit for RA diagnosis for human using ACPA from hybridoma cells [44]. This kit can provide more accurate and rapid diagnostic method for early RA patients.

As cigarette smoke is inhaled it passes through the respiratory tract at first. Interestingly, most cigarette smoke particles are deposited in the respiratory epithelium, especially the tracheal epithelium. We confirmed this phenomenon through the extraction of cigarette smoke although this extraction system is not fully realized (Fig 5B). Specifically, a closer physical proximity to the burned cigarette smoke increased the deposition of molecules like tar in the upper airway. We also confirmed changes to the extracellular matrix surrounding the tracheal cartilage in the smoking group. CIA mice exposed to smoke showed significantly higher levels of citrullination in tracheal cartilage compared with the CIA group (Fig 3A). In addition, we did not see a difference in citrullination between the CIA group and normal control group (Fig 3B–3D). Lastly, we noted that the levels of citrullination significantly varied among alveolar, bronchiolar, and tracheal tissue (Fig 3F).

Citrullinated proteins and anti-citrullinated protein antibodies play an important role in the pathogenesis and diagnosis of RA [20, 45]. We confirmed that CIA mice exposed to cigarette smoke had higher serum levels of citrullinated proteins compared to CIA mice (Fig 4B–4F). Similarly, the levels of anti-citrullinated protein antibodies were higher in CIA mice exposed to smoke compared to the CIA group (Fig 4G). Lastly, we used an *in vitro* assay combining chondrogenic pellets with PBMCs to mimic cartilage conditions. Using a prepared cigarette smoke extract (CSE) at a pre-determined concentration based on a CCK assay, we evaluated the citrullination of chondrogenic pellets. We found that citrullination was significantly higher in co-cultures of PBMCs in CSE-containing medium compared with controls (Fig 5C).

The limitations of this study include the species between human and experimental murine model, which is not fully reflected the conditions of arthritis. There are a few kinds of experimental model of arthritis, but our protocol was only optimized to mouse model, not rats or rabbits. Although, the experimental mouse model cannot completely mimic the human conditions, but its homogeneity of the genetic background can reduce individual differences.

In conclusion, we showed that cigarette smoke exacerbates arthritis in an experimental mouse model of arthritis. Cigarette smoke inhalation induced significant citrullination in various tissues, especially damaged in respiratory tract. *In vitro*, citrullination of chondrogenic pellets confirmed the effects of cigarette smoke extract on PBMCs. Taken together, our findings suggest that cigarette smoke may be an important factor in the pathogenesis of RA.

## Supporting information

S1 Fig Co-localization of enolase and citrullinated proteins. CIA group treated with cigarette smoke showed higher co-localization with enolase and citrullinated proteins than CIA group.
(TIF)

S2 Fig Determination of CSE concentration using CCK-8 assay. Above 0.5% concentration of cigarette smoke extract, there was significant difference in the cell viability.
(TIF)

S3 Fig Identification of citrullination using mass spectrometry. LC-MS/MS was used for identification of citrullination.
(TIF)

S1 File ARRIVE guidelines checklist. Animal experiments were performed according to the ARRIVE guidelines.
(PDF)
