# Supplementary figures and images for: Exacerbation of symptomatic arthritis by cigarette smoke in experimental arthritis
Source: PLoS One. 2020 Mar 27;15(3):e0230719. doi: 10.1371/journal.pone.0230719 (PMC7100974; doi:10.1371/journal.pone.0230719)

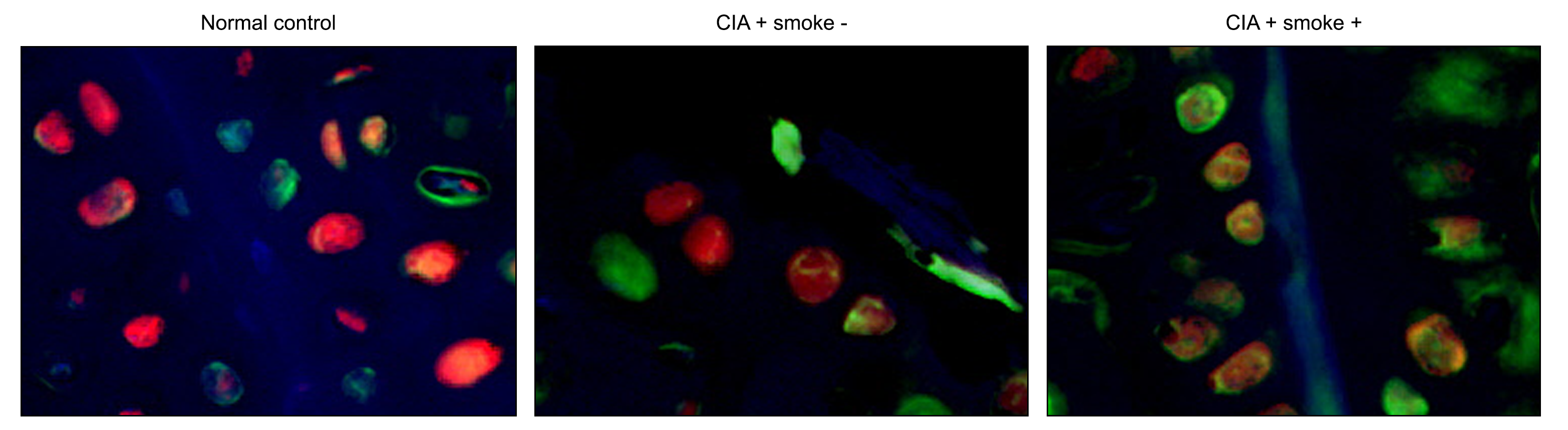

Supplement: S1 Fig — CIA group treated with cigarette smoke showed higher co-localization with enolase and citrullinated proteins than CIA group. (TIF) [file pone.0230719.s001.tif]

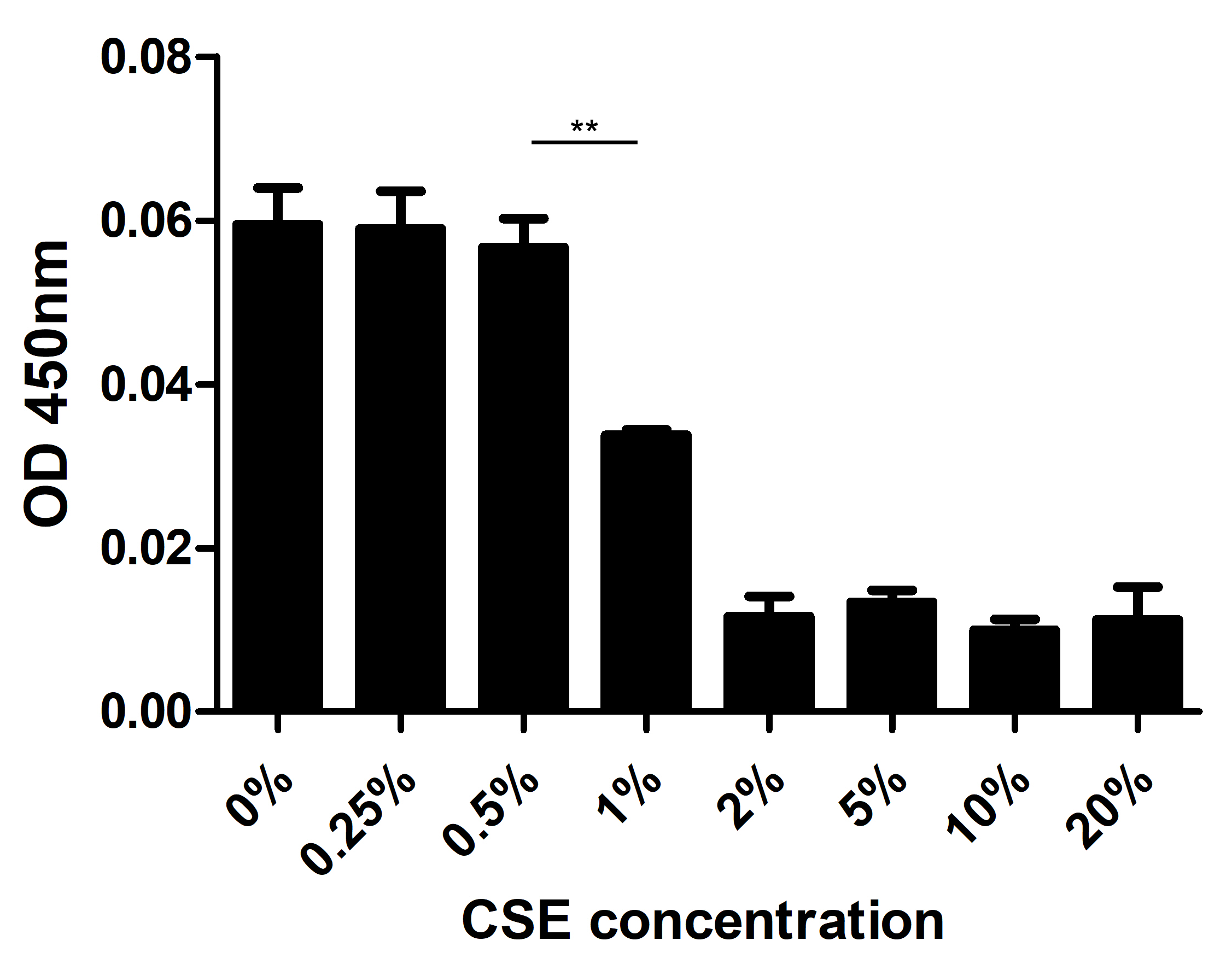

Supplement: S2 Fig — Above 0.5% concentration of cigarette smoke extract, there was significant difference in the cell viability. (TIF) [file pone.0230719.s002.tif]

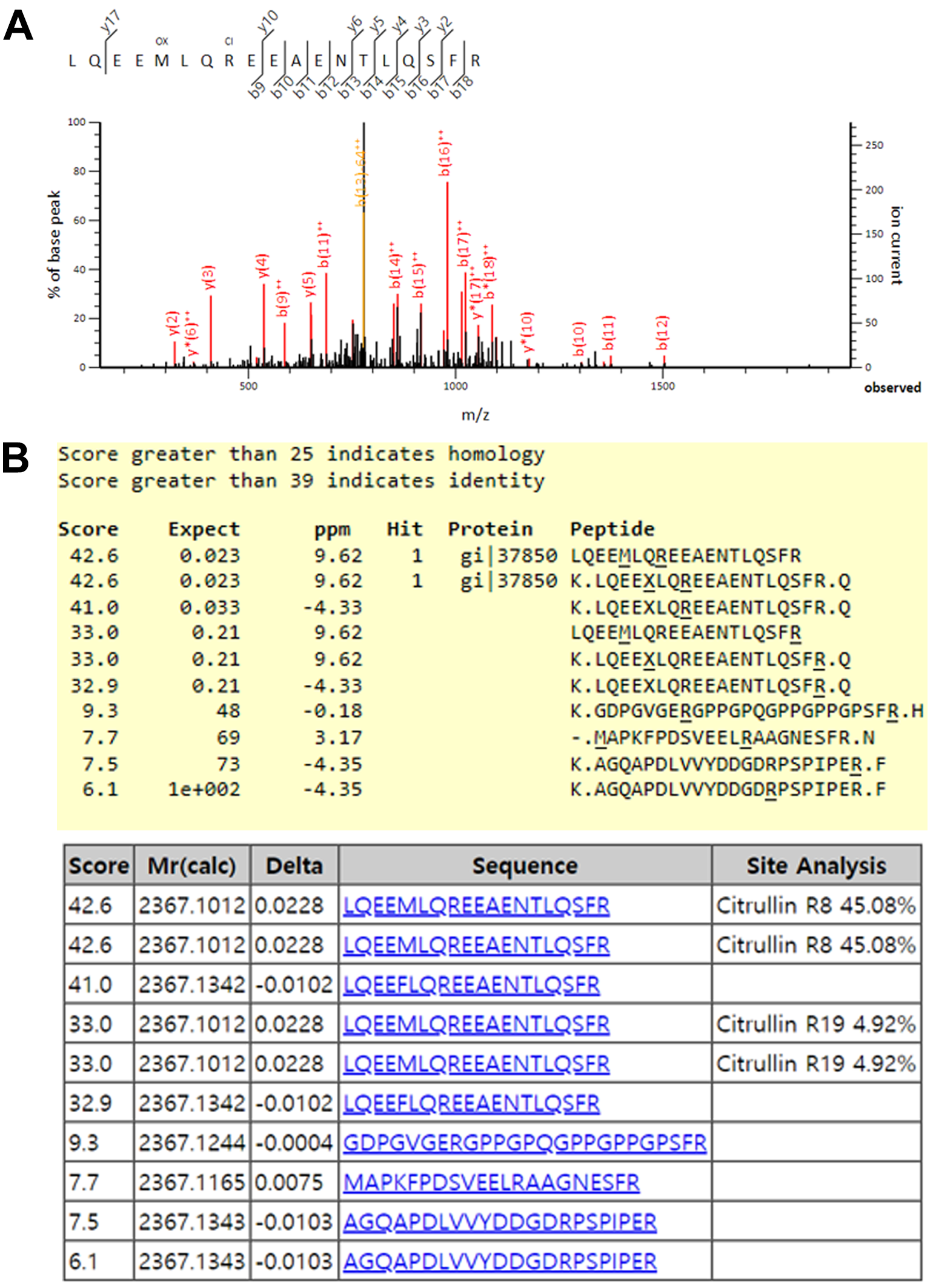

Supplement: S3 Fig — LC-MS/MS was used for identification of citrullination. (TIF) [file pone.0230719.s003.tif]
